# Co-administration of plasmid-encoded granulocyte-macrophage colony-stimulating factor increases human immunodeficiency virus-1 DNA vaccine-induced polyfunctional CD4^+^ T-cell responses

**DOI:** 10.1590/0074-02760150283

**Published:** 2015-12

**Authors:** Vinicius Canato Santana, Rafael Ribeiro Almeida, Susan Pereira Ribeiro, Luís Carlos de Souza Ferreira, Jorge Kalil, Daniela Santoro Rosa, Edecio Cunha-Neto

**Affiliations:** 1 Universidade de São Paulo, Faculdade de Medicina, Divisão de Imunologia Clínica e Alergia, São Paulo, SP, Brasil; 2 Instituto Nacional de Ciência e Tecnologia, Instituto de Investigação em Imunologia, São Paulo, SP, Brasil; 3 Universidade de São Paulo, Instituto de Ciências Biomédicas, Departamento de Microbiologia, São Paulo, SP, Brasil; 4 Universidade de São Paulo, Faculdade de Medicina, Instituto do Coração, São Paulo, SP, Brasil; 5 Universidade Federal de São Paulo, Faculdade de Medicina, Divisão de Imunologia, São Paulo, SP, Brasil

**Keywords:** granulocyte-macrophage colony-stimulating factor -, DNA vaccine, polyfunctional CD4^+^ T-cells, HIV

## Abstract

T-cell based vaccines against human immunodeficiency virus (HIV) generate specific responses that may limit both transmission and disease progression by controlling viral load. Broad, polyfunctional, and cytotoxic CD4^+^T-cell responses have been associated with control of simian immunodeficiency virus/HIV-1 replication, supporting the inclusion of CD4^+^ T-cell epitopes in vaccine formulations. Plasmid-encoded granulocyte-macrophage colony-stimulating factor (pGM-CSF) co-administration has been shown to induce potent CD4^+^ T-cell responses and to promote accelerated priming and increased migration of antigen-specific CD4^+^ T-cells. However, no study has shown whether co-immunisation with pGM-CSF enhances the number of vaccine-induced polyfunctional CD4^+^ T-cells. Our group has previously developed a DNA vaccine encoding conserved, multiple human leukocyte antigen (HLA)-DR binding HIV-1 subtype B peptides, which elicited broad, polyfunctional and long-lived CD4^+^ T-cell responses. Here, we show that pGM-CSF co-immunisation improved both magnitude and quality of vaccine-induced T-cell responses, particularly by increasing proliferating CD4^+^ T-cells that produce simultaneously interferon-γ, tumour necrosis factor-α and interleukin-2. Thus, we believe that the use of pGM-CSF may be helpful for vaccine strategies focused on the activation of anti-HIV CD4^+^ T-cell immunity.

A safe and effective human immunodeficiency virus (HIV) vaccine is still the most promising strategy for controlling the acquired immune deficiency syndrome pandemic. T-cell based vaccines generate HIV-specific responses that may limit both transmission and disease progression by controlling viral loads (Watkins et al. 2008). A recent Phase III clinical trial in Thailand (RV144) enrolling more than 16,000 individuals showed 31% efficacy in preventing viral acquisition (Rerks-Ngarm et al. 2009), which indicates that an HIV-1 vaccine is a feasible aim.

Mounting evidence suggests that polyfunctional CD4^+^ T-cell responses may be important to confer protection against *Mycobacterium tuberculosis* and*Leishmania major* challenges (Darrah et al. 2007, Lindenstrøm et al. 2009) and also for controlling HIV-1 replication (Porichis & Kaufmann 2011). Although HIV-specific CD4^+^ T-cells are preferentially targeted by the virus, the vast majority of these cells remains virus-free at any time in vivo (Douek et al. 2002), which may allow for their antiviral function. In fact, strong virus-specific CD4^+^ T-cell responses have been associated with natural control of HIV-1 infection and prediction of disease outcome (Rosenberg et al. 1997, Gloster et al. 2004, Soghoian et al. 2012). Cytotoxic CD4^+^ T-cells were shown to suppress viral replication in both simian immunodeficiency virus (SIV) and HIV-1-infected cells (Sacha et al. 2009, Zheng et al. 2009) and the frequency of polyfunctional mucosal CD4^+^ T-cells was shown to be augmented in elite viral controllers when compared to noncontrollers or individuals on highly active antiretroviral therapy (Ferre et al. 2010).

While the clinical associations of CD4^+^ T-cell responses and HIV-1 control face a cause-effect issue, the finding that CD4^+^ T-cell depletion contributes to reducing vaccine-mediated protection against SIV (Vaccari et al. 2008) supports a direct role of such cells in antiviral immunity. Also, increased polyfunctional CD4^+^ responses induced in recombinant simian varicella virus-SIV_Env/Gag_ vaccinated rhesus macaques were shown to be key correlates of vaccine-mediated protection against SIV infection (Pahar et al. 2012). Thus, inclusion of CD4^+^ T-cell targets deserves particular attention in the design of potential anti-HIV vaccine constructs.

In order to induce HIV-specific CD4^+^ T-cell responses, our group developed a DNA vaccine encoding multiple human leukocyte antigen (HLA)-DR binding HIV-1 subtype B conserved peptides (HIVBr18). We have reported that this vaccine induced broad CD4^+^ T-cell responses in mice transgenic to common HLA class II alleles (HLA-DR2, -DR4, -DQ6, -DQ8) (Ribeiro et al. 2010). In addition, HIVBr18 immunisation activated polyfunctional and long-lived central and effector memory CD4^+^ T-cells in BALB/c mice (Rosa et al. 2011). However, further improvements in HIVBr18 immunogenicity will be required before the vaccine formulation could be submitted to clinical trials.

DNA vaccine immunisation leads to direct transfection of antigen presenting cells (APCs) and tissue-resident-cells, providing local and systemic expression of target antigens and subsequent induction of cellular and humoral immunity. Professional APCs are not typically found in muscle tissue and they need to migrate to the inoculation site in response to inflammatory or chemotactic signals before an efficient immune response is mounted (Kutzler & Weiner 2008).

Plasmid-encoded granulocyte-macrophage colony- stimulating factor (pGM-CSF) has been shown to mediate the recruitment of neutrophils, macrophages and immature dendritic cells (DCs) to the immunisation site (Haddad et al. 2000) and to enhance immune responses induced by DNA vaccines in different animal models (Weiss et al. 1998, Ahlers et al. 2002, Song et al. 2006). Remarkably, it has been shown that a bicistronic DNA vaccine encoding HIV-1 gp120 and GM-CSF evoked an extensive inflammatory infiltrate and induced potent CD4^+^ T-cell responses (Barouch et al. 2002). Also, accelerated antigen-specific CD4^+^ T-cell priming and increased migration of activated CD4^+^ T-cells were observed in mice immunised with a recombinant GM-CSF-encoding BCG vaccine (Nambiar et al. 2010). However, to our knowledge no study has shown that pGM-CSF co-administration improves the quality of vaccine-induced CD4^+^T-cell responses, which is fundamental for the development of new anti-HIV vaccine approaches.

## MATERIALS AND METHODS

*Plasmids* - The plasmids used in this study were the HIVBr18 vaccine, previously developed by our group (Ribeiro et al. 2010, Rosa et al. 2011), a pGM-CSF kindly provided by Dr Sergio Costa Oliveira, from the Federal University of Minas Gerais, Brazil, as previously reported (Diniz et al. 2010), and the pVAX1 vector. The plasmids were purified using the Endofree Plasmid Giga Kit (Qiagen) according to manufacturer’s instructions.

*Peptides* - The HIVBr18-encoded peptides (Fonseca et al. 2006) were synthesized by solid phase technology using 9-fluorenylmethoxycarbonyl strategy, with the C’ terminal carboxyl group in amide form (GL Biochem, China). Peptide purity and quality were assessed by reverse-phase high performance liquid chromatography and mass spectrometry, and were routinely above 90%.

*Ethics* - Inbred BALB/c mice were obtained from the Animal Facility at the School of Medicine of the University of São Paulo (USP) and housed and manipulated under specific-pathogen-free conditions in the animal care facility at the Institute of Tropical Medicine, USP. Experiments were performed in accordance to the guidelines of the Ethical Commission for Animals Use of the USP and approved under protocol 197-12.

*Mice and immunisations* – Six-eight week-old female BALB/c mice were used in this study. HIVBr18 combined with pVAX1 or pGM-CSF was administered intramuscularly at days 0, 14, and 28. Each animal received 200 µg of plasmid combination (100 µg of each) per dose, diluted in sterile saline. The doses were divided in two injections of 50 µL, one in each quadriceps. Two weeks after the last dose, mice were euthanized in a CO_2_ chamber.

*Spleen cell isolation for immune assays* - Two weeks after the last immunisation, mice were euthanized and spleens were aseptically removed. After obtaining single cell suspensions, cells were washed in 10 mL of RPMI-1640 (Gibco, USA). Cells were then suspended in RPMI supplemented with 10% of foetal bovine serum (FBS) (Gibco), 2 mM L-glutamine (Sigma-Aldrich, USA), 10 mM Hepes (Sigma-Aldrich), 1 mM sodium piruvate, 1% vol/vol nonessential amino acid solution (Gibco), 40 mg/mL of gentamicin, 20 mg/mL of peflacin and 5 x 10^-5^ M 2-mercaptoetanol (Sigma-Aldrich). The viability of cells was evaluated using 0.2% Trypan Blue exclusion dye to discriminate between live and dead cells. Cell concentration was estimated with the aid of a Neubauer chamber and adjusted in cell culture medium.

*Detection of interferon (IFN)-γ-secreting T-cells by ELISPOT assay* - The frequency of IFN-γ-secreting T-cells was determined by incubating splenocytes (3 × 10^5^ cells/well) from immunised mice with 5 µM of individual or pooled HIVBr18-encoded peptides for 18 h at 37ºC and 5% CO_2_. The ELISPOT assay was performed using murine IFN-γ BD kit according to manufacturer’s instructions (Becton, Dickinson and Company, USA). Spots were counted using an AID ELISPOT reader (Autoimmun Diagnostika GmbH, Germany). The number of antigen-specific T-cells, expressed as spot-forming units (SFU)/10^6^ splenocytes, was calculated after subtracting negative control values (medium only). Responses were considered positive when above cut-off, which was calculated as the mean response plus 3 standard deviations (SD) of splenocytes from mock-immunised mice (pVAX or pGM-CSF only), stimulated with each peptide. A positive control was performed in all experiments by incubation of splenocytes from each immunised group with concanavalin A (ConA) (final concentration 2 µg/mL) (Sigma-Aldrich).

*Analysis of polyfunctional CD4* ^*+*^ *T-cell responses* - Briefly, freshly isolated splenocytes were suspended (5 x 10^6^/mL) in phosphate-buffered saline (PBS) and labelled with 1.25 µM of carboxyfluorescein succinimidyl ester (CFSE) (Molecular Probes, USA) at 37ºC for 10 min. The reaction was quenched with RPMI-1640 supplemented with 10% FBS. CFSE-labelled cells were incubated at a density of 2.5 x 10^6^ cells/mL and cultured in 96 well round-bottomed plates (5 x 10^5^/well) for four days at 37ºC and 5% CO_2_ with medium only or pooled HIVBr18-encoded peptides (5 µM). After four days of incubation, cells were stimulated with 2 µg/mL of anti-CD28 (BD Biosciences, USA) and 5 µM of pooled HIVBr18-encoded peptides for 1 h, and kept in the presence Brefeldin A (BD Biosciences) for additional 12 h. After the incubation period, cells were washed with fluorescence-activated cell sorting (FACS) buffer (PBS + 0.5% bovine serum albumin + 2mM ethylenediamine tetraacetic acid) and surface stained using CD4-PercP Cy5.5 and CD8-Alexa Fluor 700 monoclonal antibodies for 30 min at 4ºC. Cells were fixed and permeabilised using the Cytofix/CytopermTM kit (BD Biosciences). Permeabilised cells were washed with Perm/Wash buffer (BD Biosciences) and stained with CD3-APCCy7, interleukin (IL)-2-PE, tumour necrosis factor (TNF)-α-PECY7 and IFN-γ-APC for 30 min at 4ºC. Following staining, cells were washed twice and suspended in FACS buffer. All antibodies were from BD Biosciences. Samples were acquired on a FACSCanto flow cytometer (BD Biosciences) and then analysed using the FlowJo software (FlowJo, LLC, USA). Cells were gated on forward scatter/side scatter, CD3^+^, and CD4^+^/CD8^+^. The frequency of proliferating T-cells producing cytokines was determined by gating cells on CFSE^low^ population that were positive for IFN-γ, TNF-α, or IL-2 expression. A Boolean analysis was performed to obtain the frequency of proliferating CD4^+^ or CD8^+^ T-cells producing any combination of cytokines. As a positive control in all experiments, pooled splenocytes from each immunised group were incubated with ConA (final concentration 2 µg/mL). Background response from mock-immunised mice splenocytes (pVAX or pGM-CSF only) submitted to the same experimental conditions was subtracted from respective experimental group.

*Data analysis and statistics* - Statistical significance (p-values) was calculated by using *t* test for all experiments. The significance of single cytokine or combined cytokines produced by CFSE^low^T-cells was calculated using Student’s *t* test (since we had 2 experimental groups for each condition) and all bars were put in the same graph to facilitate visualisation. Statistical analysis and graphical representation of data were performed using GraphPad Prism 5.0 software, and p-values < 0.05 were considered significant (*: p < 0.05; **: p < 0.01; ***: p < 0.001).

## RESULTS

**Fig. 1:**
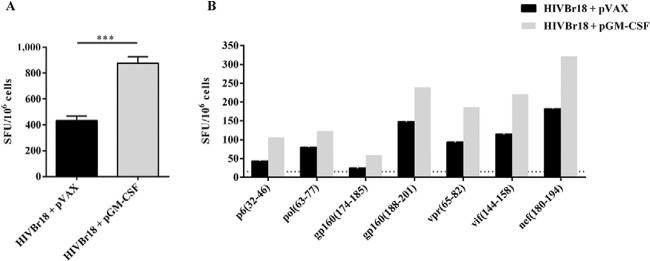
: co-administration of plasmid-encoded granulocyte-macrophage colony-stimulating factor (pGM-CSF) enhances human immunodeficiency virus-1 subtype B conserved peptides (HIVBr18)-induced T-cell responses. Two weeks after the last immunisation with HIVBr18 co-administrated with pVAX1 or pGM-CSF, pooled spleen cells from six BALB/c mice were cultured in the presence of both pooled and individual HIVBr18-encoded peptides (5 µM), or medium only. Frequencies of interferon (IFN)-γ-secreting T-cells against pooled (A) and individual (B) HIVBr18-encoded peptides were measured by ELISPOT and shown as spot forming units (SFU) per 106 cells. Mean plus standard deviation of three independent experiments are shown in A. One representative experiment of three is shown in B. Only peptides frequently recognised are shown. Dotted line represents ELISPOT cut-off (15 SFU/106 cells). Asterisks mean p < 0.001.

*Co-administration of pGM-CSF enhances HIVBr18-induced T-cell responses* - In order to evaluate whether GM-CSF would enhance HIVBr18 DNA vaccine immunogenicity, we co-immunised BALB/c mice with a pGM-CSF. As a control, we co-immunised mice with HIVBr18 DNA vaccine and an empty pVAX1 vector. We observed that co-immunisation with pGM-CSF enhanced the frequency of IFN-γ-secreting T-cells against pooled HIVBr18-encoded peptides, from 432-876 SFU/10^6^cells (Fig. 1A). To evaluate the impact of GM-CSF on the breadth of T-cell responses we measured the frequency of IFN-γ-secreting T-cells against individual HIVBr18-encoded peptides and observed that although co-administration of pGM-CSF enhanced the magnitude of T-cell responses, no difference was observed in the number of recognised peptides (Fig. 1B). Therefore, our data suggest that GM-CSF had an impact on HIVBr18 immunogenicity which was not sufficient to broaden T-cell immune responses.

**Fig. 2:**
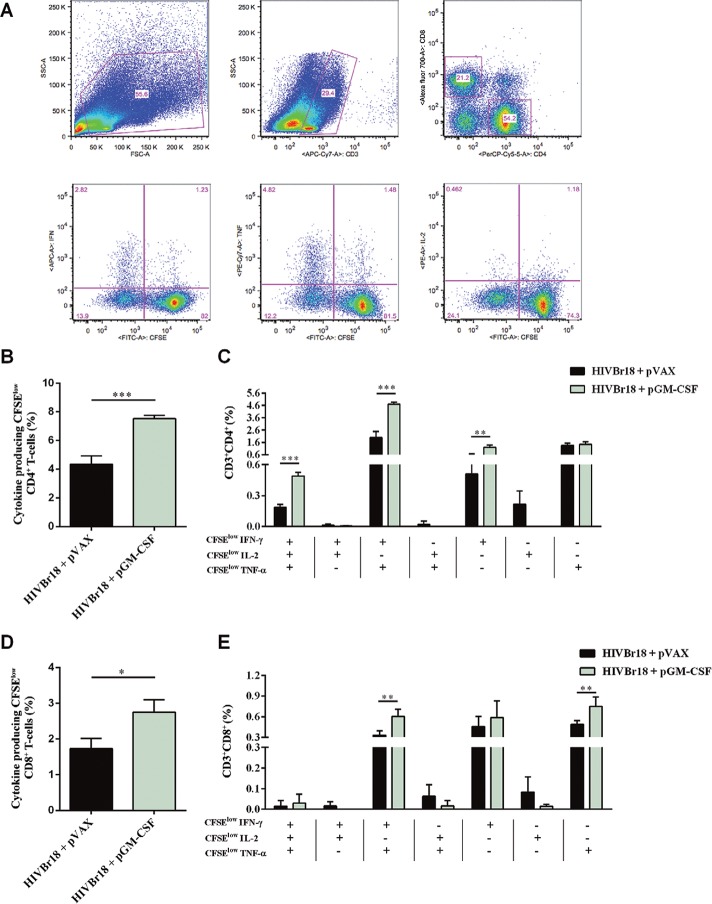
: co-administration of pasmid-encoded granulocyte-macrophage colony-stimulating factor (pGM-CSF) increases the frequency of human immunodeficiency virus-1 subtype B conserved peptides (HIVBr18)-induced polyfunctional CD4+ T-cells. Two weeks after the last immunisation with HIVBr18 co-administrated with pVAX1 or pGM-CSF, pooled spleen cells from six BALB/c mice were collected, labelled with carboxyfluorescein succinimidyl ester (CFSE) (1.25 mM) and cultured for four days in the presence of pooled HIVBr18-encoded peptides or medium only. On day 4, cells were pulsed for 12 h with pooled peptides in the presence of Brefeldin A and co-stimulatory antibody (anti-CD28), and then stained for CD3, CD4, CD8, interferon (IFN)-γ, tumour necrosis factor (TNF)-α, and interleukin (IL)-2. Multiparameter flow cytometry strategy used to determine the frequency of polyfunctional T-cells (A). Frequency of cytokine producing CFSElow CD4+ T-cells (B) and CD8+ T-cells (D). Frequency of CFSElow CD4+ (C) and CD8+ (E) T-cells producing all the combinations of IFN-γ, TNF-α, and IL-2. Background responses were subtracted in each condition. One representative experiment of three is shown in B-E. APC: antigen presenting cell; FITC: fluorescein isothiocyanate; FSC: forward scatter; SSC: side scatter; *: p < 0.05; **: p < 0.01; ***: p < 0.001.

*Co-administration of pGM-CSF increases the frequency of HIVBr18-induced polyfunctional CD4* ^*+*^ *T-cells* - We also addressed the question whether GM-CSF would improve the quality of HIVBr18-induced immune responses by measuring the frequency of antigen-specific polyfunctional CD4^+^ T-cells. We immunised BALB/c mice as previously mentioned and evaluated the frequency of CD4^+^ or CD8^+^ T-cells that were both proliferating and producing the cytokines IFN-γ, TNF-α, and IL-2 (Fig. 2A). We observed that co-administration of pGM-CSF increased the frequency of proliferating CD4^+^ T-cells producing any cytokine against pooled HIVBr18-encoded peptides, from 4.33-7.52% (Fig. 2B). In order to determine whether the proliferating CD4^+^ T-cells were single cytokine producers or polyfunctional cells, we analysed the ability of those cells to proliferate and produce all possible combinations of IFN-γ, TNF-α, and IL-2. We observed that co-administration of pGM-CSF significantly improved the frequency of proliferating CD4^+^ T-cells producing IFN-γ (from 0.5-1.2%), IFN-γ, and TNF-α (from 1.98-4.70%), or all three cytokines (from 0.18-0.49%) (Fig. 2C). Similarly to CD4^+^ T-cells, co-administration of pGM-CSF increased the frequency of proliferating CD8^+^ T-cells producing any cytokine, from 1.73-2.75% (Fig. 2D). On the other hand, we observed that co-administration of pGM-CSF significantly improved the frequency of proliferating CD8^+^ T-cells producing, exclusively, TNF-α (from 0.48-0.75%), IFN-γ and TNF-α (from 0.33-0.60%), and no difference was observed among CD8^+^T-cells producing all three cytokines (Fig. 2E). Thus, our data indicate that GM-CSF enhanced the frequency of HIVBr18-induced polyfunctional CD4^+^ T-cell responses.

## DISCUSSION

In this study, we have shown that co-administration of pGM-CSF and the DNA vaccine HIVBr18 improved both the magnitude and the quality of antiHIV-1 T-cell immune responses, particularly by increasing the frequency of polyfunctional CD4^+^ T-cells displaying proliferation and capable to secrete different cytokines (IFN-γ, TNF-α, and IL-2). Natural or vaccine induced CD4^+^ T-cells populations can exert wide effects, for example, direct cytotoxic effect on infected cells (Sacha et al. 2009, Zheng et al. 2009). Also, it can provide co-stimulatory signals to APCs for the activation and induction of effector and memory CD8 T-cell populations (Arens & Schoenberger 2010). Those functions are dependent of cytokine profile production, and in particular for HIV-1 infection, a better quality and protective immune response can be sustained if those polyfunctional T-helper 1 CD4^+^ T-cell are present (Ferre et al. 2010)*.*

The finding that co-administration of a plasmid encoding GM-CSF enhanced DNA vaccine immunogenicity, measured by the frequency of antigen-specific IFN-γ-secreting T-cells, is in accordance with previous evidence (Barouch et al. 2002, Nambiar et al. 2010). In fact, the role of GM-CSF as a vaccine adjuvant has been extensively studied, showing that both T and B-cell immune responses may be improved. Locally recruited antigen-presenting cells were shown to be critical for the magnitude and nature of such responses (McKay et al. 2004, Robinson et al. 2006,Qiu et al. 2007). Furthermore, intramuscular GM-CSF co-administration has been shown to increase the number of infiltrating CD11c^+^ DC) and splenic DCs, expression of major histocompatibility complex class II on splenic DC, and enhance the antigenic capture, processing and presentation functions of splenic DCs (Zhai et al. 2015).

Here, we have shown that although the magnitude of HIVBr18-induced T-cell responses against both pooled and individual peptides was augmented by the co-administration of pGM-CSF, no difference in the breadth of epitopes recognised by T-cells was observed. As far as we know only one study has explored the impact of GM-CSF on the breadth of T-cell responses induced by vaccination (Rodríguez et al. 2012). In this case, it was shown that pGM-CSF co-administered to DNA-prime followed by modified Vaccinia Ankara-boost broadened T-cell responses to pooled peptides representing five different HIV-1 Nef domains. Such results could not be reproduced in mice immunised with HIVBr18, which encodes isolated peptides previously selected by the potential immunogenic profile to CD4^+^ T-cells (Ribeiro et al. 2010). In fact, the multiepitope vaccine approach was originally designed to overcome immunodominance effects leading to broad epitope recognition *per se* (Livingston et al. 2002).

The fact that our DNA vaccine was rationally designed for inducing anti-HIV-1 CD4^+^ T-cell immune responses (despite having internal CD8 recognition epitopes) led to the question whether co-administration of pGM-CSF would change the immune responses mediated by those cells. We found that the frequency of polyfunctional CD4^+^ T-cells was significantly enhanced in mice co-immunised with HIVBr18 and pGM-CSF, particularly those producing IFN-γ and TNF-α, or IFN-γ, TNF-α, and IL-2.

Our results demonstrated that, besides enhancing the magnitude of CD4^+^ and CD8^+^ T-cell responses to a co-administered DNA vaccine-encoded antigen, expression of GM-CSF had a significant impact on the activation of CD4^+^ T-cells capable to secrete different cytokines after antigen-specific stimulation. It has been described that immunisation with a bicistronic plasmid co-expressing HIV-1 gp120 and GM-CSF under control of a single promoter resulted in a dramatic augmentation of both vaccine-induced proliferating and IFN-γ-secreting CD4^+^ T-cells (Barouch et al. 2002). Also, a vaccine based on a BCG encoding GM-CSF led to accelerated antigen-specific CD4^+^ T-cell priming and increased migration of activated CD4^+^ T-cells into the lung, resulting in significantly increased protection against *M. tuberculosis* (Nambiar et al. 2010).

The ability of GM-CSF to improve the frequency of polyfunctional T-cells was previously described for CD8^+^ T-cells. It was shown that co-expression of GM-CSF and ovalbumin (OVA) in a DNA-prime adenoviral-boost immunisation resulted in a striking expansion of polyfunctional OVA-specific CD8^+^ T-cells (Tenbusch et al. 2008). Moreover, the use of pGM-CSF as an adjuvant for DNA vaccines expressing HIV-1 Gag and Nef-Tat-Vif increased the frequency of antigen-specific polyfunctional memory CD8^+^T-cells (Xu et al. 2008). Co-expression of GM-CSF has also been shown to increase the titres and antigen avidity of antibodies, and antibody-dependent-cellular cytotoxicity induced by a DNA vaccine encoding six SIV proteins (Lai et al. 2011). However, that study failed to show any improvement in the frequency of DNA vaccine-induced polyfunctional CD4^+^ T-cells.

In our study, co-administration of pGM-CSF and HIVBr18, which encodes multiple HLA-DR binding HIVBr18 peptides, increased the frequency of polyfunctional CD4^+^T-cells as measured by antigen-induced proliferation and concomitant expression of IFN-γ, IL-2, and TNF-α. Although the specific mechanisms driving the GM-CSF-mediated enhancement of antigen-specific polyfunctional CD4^+^ T-cells activation have been not elucidated, we believe that this new finding may contribute to the development of DNA vaccines focused on inducing anti-HIV CD4^+^ T-cell immunity.
